# Primal Eukaryogenesis: On the Communal Nature of Precellular States, Ancestral to Modern Life

**DOI:** 10.3390/life2010170

**Published:** 2012-01-23

**Authors:** Richard Egel

**Affiliations:** Department of Biology, University of Copenhagen Biocenter, Ole Maaløes Vej 5, DK-2200 Copenhagen, Denmark; E-Mail: regel@bio.ku.dk; Tel.: +45-4589-3746; Fax: +45-3532-2128

**Keywords:** origin of life, molecular ecosystems, protoeukaryote-early concept, *Karyogenic Proto-Coenocyte Hypothesis*, hierarchical clonality, pre-cell theory, cellular escape, *K*-selection, *r*-selection

## Abstract

This problem-oriented, exploratory and hypothesis-driven discourse toward the unknown combines several basic tenets: (i) a photo-active metal sulfide scenario of primal biogenesis in the porespace of shallow sedimentary flats, in contrast to hot deep-sea hydrothermal vent conditions; (ii) an inherently complex communal system at the common root of present life forms; (iii) a high degree of internal compartmentalization at this communal root, progressively resembling coenocytic (syncytial) super-cells; (iv) a direct connection from such communal super-cells to proto-eukaryotic macro-cell organization; and (v) multiple rounds of micro-cellular escape with streamlined reductive evolution-leading to the major prokaryotic cell lines, as well as to megaviruses and other viral lineages. Hopefully, such nontraditional concepts and approaches will contribute to coherent and plausible views about the origins and early life on Earth. In particular, the coevolutionary emergence from a communal system at the common root can most naturally explain the vast discrepancy in subcellular organization between modern eukaryotes on the one hand and both archaea and bacteria on the other.

## 1. Preface

Life on Earth represents the highest level of complexity attained on this planet. Individual organisms exist on borrowed time, on the verge of dissipation, but the collective continuity of living matter has lasted several gigayears (Ga). How could this self-propelling organic system ever have come about from inorganic sources on the pristine Earth? There is no easy or simple shortcut to grasping the gist of life's emergence, and certain preconceptions about the presumptive nature of the *missing link* seem overly simplistic. Assuming some kind of *simplest cell* at the primeval junction may be insufficient and potentially misleading. While *simple-cell* models tend to overemphasize the importance of secluded space at a tiny scale, I will here argue for the significance of openness and material exchange over considerable distances-all the way from geochemical flow-type reactors to a precellular organic matrix of coenocytic (syncytial) properties.

To narrow down the problem of how life originated, there are two complementary approaches-referred to as *Top-down vs. Bottom-up* analyses. Working forward (up) from tentative early-Earth conditions, inferences based on geochemical principles aim at sorting out how certain possible reactions could eventually win over ever so many others in launching a self-organizing evolutionary cascade. Conversely, extrapolating backward (down), from present *life as we know it*, researchers can point out ancient ancestral stages, but cannot precisely reconstruct the evolutionary course or phenotypical detail of all the interactive players in the distant past. Connecting the loose ends from forward-up and backward-down approaches to a coherent story is still a formidable challenge. Rethinking of cherished metaphors and paradigms is called for at various levels.

**Figure 1:**
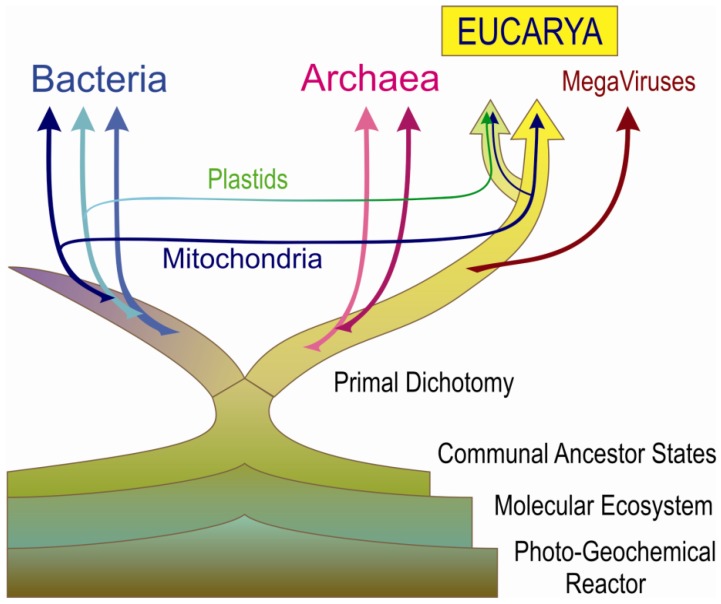
Recasting the early phylogenetic *tree of life*. The diagram emphasizes an unbroken chain of communal evolution from photo-geochemical reactors and organic molecular ecosystems to complex eukaryotic macro-cells. From the common matrix of communal ancestor states (here conceived as syncytial-like super-cells), prokaryotic micro-cells and acellular viral lineages "escaped" multiple times, as based on partly overlapping sampling of unified genomes from a highly redundant communal *gene pool* and subsequent clonal propagation. The *primal dichotomy* between bacterial and archaeal/protoeukaryotic stemlines occurred at the level of communal ancestors. All the composite modern eukaryotes descend from an ancestor that had already adopted some fairly advanced bacteria as permanently integrated mitochondrial endosymbionts. Similarly, cyanobacterial endosymbionts were acquired as plastids by the diverging lineage of green plants.

In major parts of this hypothesis-driven essay (Sections 5,6,7,8) I will focus on the upper reaches of the conceptual gap, shortly before evolutionarily stable cellular organisms emerged from less distinctively definable precursory stages. This view implies that the most basic features of eukaryotic cell organization are more representative of a communal precellular ancestral state (having coenocytic/ plasmodial characteristics at large) than is any decidedly bacteria-like prokaryotic side line. Eukaryotic and prokaryotic lineages have presumably derived from the communal ancestral state independently by following evolutionary trends that were constrained quite differently. The summarizing diagram (Figure 1) underscores this punchline, the full significance of which becomes apparent later on (Section 8). In particular, the origin of eukaryotic nuclei is conceived according to the *karyogenic hypothesis* from within a progressively syncytial-like precellular matrix (Section 7.2), which is not represented in the schematic branching pattern of the drawing.

To start with (Sections 2,3,4), and to relate this evolutionary scenario of eukaryogenesis to earlier prebiotic stages, I will point out some current highlights from the forward-up approach to primordial biogenesis, including the potential of mineral-assisted photosynthesis. It is a recurring theme that a vast richness of combinatorial possibilities can be successively narrowed down to rather few cooperative interactions which, in turn, support and drive the collective network system as a whole.

To specify my use of terms-and chemically oriented colleagues might not agree with this broadened vocabulary concept-I am looking at the scene of origins from the perspectives of molecular and cell biology, from which I rather loosely apply *primordial biogenesis* and *primordial life* to the entire evolutionary period from the first organic syntheses in a mineral world to the first emergence of full-fledged, autonomously propagating cells from a communal precellular matrix. Similarly, *primordial traits* is used in a relative sense, as going back to before the last common ancestor of the particular group in question.

## 2. The Photoactive Metal Sulfide Scenario

*"Will we ever and, indeed, do we have to know the historical path of chemical evolution? ... We wish to satisfy our inborn sense of curiosity and our desire for universal knowledge of this world. However, surprisingly, the answer to both questions is '**No**' "* [1]. This said, it is a valid scientific goal to aim at converging on some model sequence of chemically and thermodynamically possible events-how life can have emerged in general terms. For that matter, Follmann and Brownson [1] present a profoundly referenced discourse on currently prevailing views and their historical development, as seen from physico-chemical bottom-up perspectives. Yet, additional facets and reactions are needed to satisfactorily bridge the remaining gaps between bottom-up and top-down considerations to rationalize putative origins of organismal life [2,3,4].

Most significantly, according to a putative *Zinc World* concept [5,6], a suitable engine for driving the reductive tricarboxylic acid (rTCA) cycle has been found in sunlight-promoted photoactivation of mineral metal sulfide (MeS) catalysts [7,8,9,10]. This *photoactive MeS scenario* adds credibility to the assumption of prebiotic protometabolic networks, where the rTCA cycle plays a central role [11,12,13]. From the rTCA cycle, essentially all the small organic ingredients of living matter can be derived in relatively few side reactions: carbohydrates, terpenoids and fatty acids on the one hand, amino acids, nucleobases and other heterocyclics on the other. Many of these reactions need catalytic aids and/or additional energy input for proceeding at appreciable rates. Hence, the emergence of self-confining, self-enforcing and collectively system-supportive networks of mineral/organic catalysts is considered critical for the early phase of prebiotic evolution [14,15]. These geochemical networks can be conceived as prebiotic *molecular ecosystems* [16]. Yet more generally, the phenomenon of self-perpetuating life appears to be embedded in a universal *self-organizing fractal theory* [17], in accordance with *nonequilibrium thermodynamics*, where intricate electron transport networks couple molecular reaction pathways to solar-terrestrial redox gradients.

The photosynthetic activity of simple and abundant minerals, such as ZnS and MnS, is highest in the UVB part of the solar spectrum [9], which on the early Earth was not yet filtered out by any ozone layer in the virtually anoxic pristine atmosphere. Thus, as suggested long ago [18], sunlight could actually provide an ample prebiotic source of convertible energy. This insight moves the likely cradle of life back up to active surfaces-close to the interactive triple junction of lithosphere, hydrosphere and atmosphere. Such environments are fluctuating at various scales-much in contrast to deep-sea hydrothermal vents, which are currently advocated as likely sites for where and how primordial life may have emerged [19,20,21,22]. Fluctuating environments at moderate temperatures are indeed considered favorable prerequisites for biogenesis, especially when it comes to multiple condensation reactions, so as to form macromolecular associations upon dehydration. Wet/drying cycles, in particular, have most frequently been referred to for such potential [23,24,25,26,27,28,29,30]. Showing similar effects, freeze/thawing cycles are likewise worth mentioning in this context [31,32].

As for the putative self-organization of prebiotic molecular ecosystems of self-confining auto- and cross-catalytic networks, various models of an *RNA-world* scenario maintain a central role in current understanding [33,34,35], as supported by *top-down* and *bottom-up* inferences [36]. Indeed, it is now widely accepted that RNA preceded DNA as a replicatable repository of sequence information, and that the establishment of ribosome-directed synthesis of RNA-encoded proteins took place at the *RNA-world* level. It is not at all clear, however, how self-sufficient RNA (as posited by a stringent *RNA-first* model) can have emerged from geochemically sound precursory conditions [14,37]. Moreover, the key role of functional RNA in primordial biogenesis is hardly compatible with hypothetical hot-start scenarios for self-maintaining life [38].

Certain RNA molecules can, in fact, express amazing catalytic potential, but such contemporary ribozymes primarily effect the processing of RNA itself. Intermediary metabolism, on the other hand, is solely managed by protein enzymes, as assisted by transition metal complexes and various heterocyclic organic cofactors. Thus it is more reasonable to assume that a multitude of heterocyclic coenzymes already existed beforehand, so as to organize a coherent protometabolism in a primal *cofactor-world* scenario [39,40,41], well before the establishment of polymeric RNA.

Not inconceivably, these primordial heterocyclic coenzymes coexisted and cooperated with oligomeric peptide-like amino acid condensates of more or less stochastic composition [25,28,42]. In terms of (proto-)metabolic availability, many amino acids are more readily derived from the rTCA cycle than any heterocyclic compounds, including the nucleic bases as RNA precursors. In present life, in fact, both pyrimidine and purine moieties themselves are synthesized from various amino acids. Hence, there is no reason to discount the generation of prebiotic peptides as being irrelevant in comparison with RNA, especially if prebiotic mechanisms were available to chemically activate amino acids for peptide bond formation. Notably, several amino acids-assumed to be most abundant under early-Earth conditions-can form simple motifs that are able to bind metal ions and/or phosphate groups, and which still occur in many enzymes [43,44].

Moreover, simple aliphatic peptides are prone to self-aggregation in membrane-like assemblies [45], which is considered highly relevant to the primeval self-organization of lipoid/water interfaces [46,47,48]. Such surface properties render peptides complementary to RNA in many ways, greatly facilitating the interactive coevolution of both types of oligomeric chains from very early beginnings [49]. Their contrasting properties are deeply rooted in structural organization, owing to different distribution of hydrogen-bonding capacity vs. hydrophobic epitopes in either class. To summarize the complementary advantages of peptides/proteins *vs*. nucleic acids, proteins are much more versatile and expeditious as catalysts for most types of biochemical reactions, while both RNA and DNA excel in information storage and faithful replicatability [36]. Furthermore, aliphatic peptides and proteins are very good at organizing cellular and intracellular compartments, while nucleic acids are devoid of such capability. In particular, it takes the specific binding potential of intervening proteins to tether RNA or DNA to biomembrane surfaces. Last, not least, it is RNA itself that universally activates amino acids in the transfer reaction of tRNA-catalysed and ribosome-mediated protein synthesis (Section 3).

Even uncoded, stochastic peptides should be subject to self-organizing, selective evolution. There are two important facets to this intriguing possibility, depending on differential rates of copolymer formation or depolymerization, respectively. Most widely cited, models of "*catalytic closure*" [50,51,52] assume system-wide cross-catalytic production of a limited number of peptides, as preferentially amplified from a considerably larger set of possible varieties in combinatorial sequence space. Potentially of more importance yet, differential breakdown rates can likewise result in the selective survival of a limited number of hydrolysis-resistant sequences [53]. In this case, the system-wide "usefulness" of preferentially retained subsets relates to the fact that catalytic activities of any kind might be more likely to arise in spatially constrained peptide structures, rather than in random-coil configurations. Conversely, random-coil peptides tend to be more susceptible to hydrolysis, as compared with tightly constrained structures. Such cooperative stabilization can be brought about by hydrophobic interactions, leading to aggregation between different peptide sequences or between peptides and specifically binding substrates. Either kind of interaction can decisively contribute to evolutionary self-organization of catalytic surfaces. The hydrophobic interactions between lipophilic peptides, in particular, are here considered of paramount importance in the successive internalization of photosynthetic reaction centers from MeS mineral grains into the gradually complexifying organic hydrogels (Section 4.4).

## 3. The Phospho-Riboside Connection

No subcellular entities manifest the coevolution of RNA and peptides/proteins better than ribosomes, where gene-encoded proteins nowadays are made. Yet, at the putative origins of ribosome evolution, the emphasis was not yet on sequence decoding, but merely on amino acid oligomerization as such. Indeed, the peptidyl transferase center is recognized as the evolutionarily oldest core of the ribosome, assumed to have facilitated the synthesis of uncoded peptides to begin with [54,55].

More often than not, the ribosome is considered a bona fide ribozyme, which is not fully justified. Rather, the transpeptidation reaction is better characterized in terms of *substrate-assisted catalysis* [56], in which the ribosomal RNA is not engaged directly. Both the nascent peptide and the incoming next amino acid are linked by high-energy bonds to terminal ribose moieties at their respective tRNA adaptors, and the catalytic function of transpeptidation has been ascribed to the vicinal OH-group of the peptide-bearing ribose [57]. This leaves the ribosome in the role of a sophisticated scaffold superstructure, acting very much as a mechanical ratcheting device to ensure processivity of several reaction cycles in a row [58,59]. For most of its work cycle, in fact, the ribosome forms a particularly unreactive shield, so as to protect the energy-rich peptide-bearing ester bond from accidental hydrolysis by ambient water molecules [60]. Contrary to most other catalysts, however, the ribosome itself does not form any reactive intermediate with the substrates, since the meta-stable transition structure in the peptidyl transferase reaction is formed between the tRNA substrates themselves [56]. Hence, to maintain the ribosome's status as a ribozyme in a broader sense, Agmon *et al.* [61] use '*positional catalysis'* for the ribosomal mechanism, as distinguished from the more familiar '*chemical*' type.

It is the versatile reactivity of ribose itself, apparently, that conceptually connects the generation of quasi-stochastic peptides on the one hand, and heterocyclic ribotide cofactors in general on the other-including the first pyrimidine and purine derivatives. In its multiply phosphorylated form PRPP (5’-phosphoribosyl 1’-pyrophosphate), ribose is a common precursor for RNA as such and all the metabolically important ribotide coenzymes that are never incorporated into polymeric RNA. If ribose phosphates, such as PRPP, already were available in a pre-RNA protometabolic ecosystem, these could directly have served as amino acid activating agents, suited to generate small stochastic peptides spontaneously. Making longer peptides efficiently, however, required protective support from rigid scaffolds, large enough to surround the nascent peptides from all sides, such as polymeric RNA in the emerging protoribosomes.

## 4. Molecular Ecosystems in Biogenic Photochemical Reactors

While much of the literature on origins of life is guided by the conviction that early cellularization from tiny vesicles, as followed by subsequent complexification from within, provides the soundest basis for designing relevant model experiments [62,63,64], this is not necessarily the most plausible solution to life's enigmatic emergence on the pristine Earth. From an integrative alternative perspective I argue for an ecosystem-centered concept, which eventually allows individualizing subsystems to "*escape*" quite naturally, yet at a fairly advanced level of internal cellular complexity. This view leans toward progressive modularization with widespread communal interactions, where cell-like encapsulation comes in relatively late, and at high levels of hierarchical modularity. In particular, I envisage Darwinian evolution to emerge in successive stages of *hierarchical clonality*: at first, replicatable molecules begin to compete with other replicators in a common supportive matrix; next, functional compartments begin to compete with other compartments-still being surrounded by a common supportive matrix; only later on can genetically independent cells emerge and propagate with a reasonable chance of surviving as a clonal lineage, in competition with other clonal cell lines in a surrounding ecosystem.

### 4.1. Recasting the Plot

The making and propagative maintenance of "*The First Cell*" must have resulted from successful merging of (at least) three fundamental capabilities into a single physical entity: "*to copy informational macromolecules, to carry out specific catalytic functions, and to couple energy from the environment into usable chemical forms*" [65]. However, the simultaneous "*invention*" of all three (or more) achievements in the same vesicle-like confinement of space to "*jump-start*" life would remain a rather remote and scientifically inapproachable-*miraculous*-event, had not the ambient system of prebiotic interactions become increasingly robust beforehand. Not inconceivably, that is, molecular ecosystems could complexify substantially without persistent dispersion into physically maintainable subsystems of individualizable and clonally propagative identity.

To paraphrase this inbuilt tendency of system-wide modularity and complexification, the key features mentioned above and others would repeatedly be reinvented by stochastic nucleation and with accelerating frequencies. If and when these "reinventions" occurred frequently enough to cluster all these features in close proximity to one another many times, it became "useful" to invent additional measures, such as coordinated segregation mechanisms and regular division, so as to retain the multiple components that master the crucial achievements in the same physical compartment.

First of all, I should prefer to rearrange the main items in a more natural order:

(i) to couple energy from the environment into *usable* chemical forms;
(ii) to carry out *specific* catalytic functions;
(iii) to make and/or copy macromolecules;
(iv) to give some of these *informational* significance.

None of these primal capabilities as such requires vesicular inclusion to be effective at a system-wide level. Also, the three categories are not mutually independent, as designated by their respective qualifiers (in italics), which are only meaningful in a collective, system-wide context.-Why this, not that?-It is the incremental channeling into *usable*, *specific* and *informational* interrelationships-discriminating against plentiful side reactions devoid of such networking potential-that biogenic self-organization is all about.

### 4.2. A Porespace Setting in Shallow Sediments

**Figure 2:**
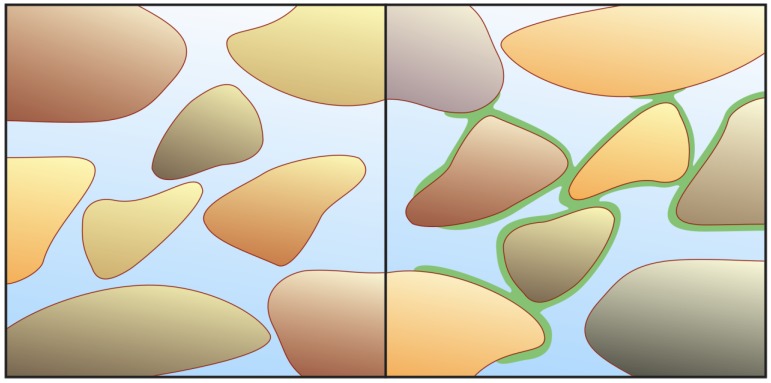
Porespace and mineral-facilitated biogenesis. In the multiply connected porespace between sedimented mineral grains (left panel), water can flow in various directions. Confluent layers of biogenic organic hydrogels (right panel, green) accumulate and progressively cover much of the mineral surface, whilst water can still move back and forth through most channels in the porespace network. The organic matrix does not only spread by incremental growth, but also by turbulent currents that redistribute the upper layers of the sediment. This diagram emphasizes the connectivity throughout a contiguous porespace. It does not, however, represent the nanoscale roughness and heterogeneity of the catalytically important mineral grains.

A common mantra is that pristine vesicles are needed early on to avoid the dilution of vital components into the open sea, but the bulk of the ocean or exposed surfaces at a rocky promontary are not the most likely settings for emergent life to begin with. Rather, the high surface to volume ratio in vast extents of porespace between mineral grains (Figure 2) provides more suitable conditions [66,67,68,69], be this in precipitating aggregates at hydrothermal vents or on sedimentary, coastal/riverine mud flats or silty banks. For photo-energetic considerations, as mentioned above, I give precedence to the latter alternative. The edge and wedge zones of weathering micaceous grains, in particular, appear especially suited for mineral-facilitated organic reactions [69].

The biogenic potential can be conceived in terms of large-scale flow bed reactors, where surface-adsorptive properties prevail-rather than as batch processing of solute reactions in closed vessels. Hence, vesicular containment should not be an essential prerequisite early on-not even for energetic reasons (see below). To colloquially distinguish such porespace setting from less likely "primordial soup" or "warm pond" scenarios, it has been referred to as "*primordial pizza*" [70] or a "*warm mud pie*" [65]. As for the local consistency of biogenic layers spread out on solid surfaces, phase-separated hydrogels were supposedly dominated by macromolecular crowding, well before the generation of autonomous cell-like entities [71,72,73,74]). The continuity of such *proto-cytoplasmic hydrogels* appears to be pivotal all the way toward full-fledged life. Coevolving with these self-cohesive organic matrix layers, micellar and membrane-like associations could not only serve as external boundaries, but also form various invaginations and other kinds of internal substructure [75].

### 4.3. Catching and Utilizing Photons by Photoactive Minerals

The flat-bed geochemical reactors envisaged here were spread out in a polarized field, with solid grounds underneath, and solar UV radiation entering the uppermost sediment layers of fine-grained to silty water-soaked minerals from above. Flow patterns responded to shifting currents and intermittent desiccation. Longer periods of alternating laminated flow in the porespace of settled sediments were occasionally disturbed by turbulent redistribution in major portions of the reactor bed. Hence, stratifying and recycling trends could have been superimposed on one another.

The stratification of organic molecules in such a primordial reactor was mainly driven in response to unidirectional illumination. In terms of chemical effects, the energetic UV light was both a blessing and a curse. Together with MeS minerals, UVB rays could drive the rTCA cycle of primal organic syntheses [5,7,9], the primary source for most other organic compounds and reactions [11]. On the other hand, their energy is sufficient to break covalent organic bonds quite easily.-How then could larger biogenic molecules ever have formed under such harsh conditions?

'*Survival of the fittest*' may indeed have begun quite early, selecting for the highest level of *photostability* among various kinds of organic compounds. All organic bonds are not created equal in this regard. Hydrogen-bonded complexes of aromatic π systems, such as base-paired RNA and DNA, are remarkably resistant against UV-induced decomposition [76,77]. In response to UV photons absorbed in the ring systems, the intimate coupling of vibrational resonance at different levels allows the energy input to be rapidly dissipated via the hydrogen bonds as heat to the environment-usually well before any covalent bond can be broken. By the same token, the intramolecular hydrogen bonding in peptide/protein secondary structure, such as α-helixes and β-strands or β-sheets, is likewise capable of efficient energy dissipation [78], which may protect the potentially fragile peptide bonds.

Accordingly, the first RNA-like biopolymers can have accumulated early on as exceptionally UV-photostable organic compounds [79], together with aggregated peptides and other hydrogen-bonded polymers. The interactive gelling of RNA with simple amyloid-like peptides suggests itself as a relevant model reaction in this context [80,81]. In the porespace of biogenic silty banks envisioned here, such polymers would thus conglomerate in a distinct matrix layer at a certain depth of the sediment, somewhat below the zone of maximal MeS-dependent photosynthesis. Below this protective sunshade layer in the UV-range, other delicate biogenic processes could proceed more safely, including the RNA-mediated polymerization of longer stochastic peptides (see above) and a possible shift to longer-wavelength photosynthesis, as facilitated by organic pigments in hydrophobic pockets.

**Figure 3:**
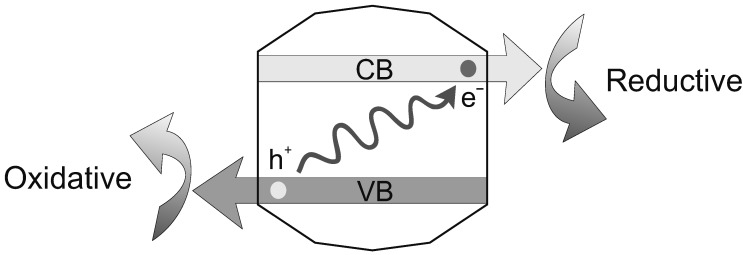
Photochemical charge separation, coupled to redox reactions. In a colloidal MeS particle (center), absorption of a UVB photon mobilizes an *electron* (e^-^) of the crystalline lattice into the energetic *conductance band* (CB). This leaves a complementary void in the lattice, which is conductable, too, as a *hole* (h^+^) in the *valence band* (VB). Both these 'bands' represent different levels on an energy scale. Upon reaching the particle surface, e^-^ and h^+^ can initiate *reductive* and *oxidative* biochemical reaction pathways, respectively. Figure modified from Refs. [9,82].

The important aspect of energy harvesting at membrane-bounded compartments can have evolved in several steps from simple beginnings. The coupling of organic syntheses to inelastic absorption of UVB photons by MeS minerals (as mentioned above) is facilitated by a colloidal consistency of the photoactive particles [9,82]. The primal act of photoactivation is the simultaneous generation of an energetic pair (Figure 3), consisting of a *conductance-band electron* (e^-^) and a complementary *valence-band hole* (h^+^), both of which can readily migrate through the crystalline lattice. At the nanoscale of collodial particles, both e^-^ and h^+^ have a reasonable chance of reaching the surface, where they can be scavenged in *reducing* and *oxidizing reactions*, respectively-rather than being annihilated by instantaneous *charge recombination* and liberating heat within the mineral grain itself.

### 4.4. Membrane Compartmentalization of Charge Separation

The residual risk of unproductive charge recombination close to the colloidal particle could diminish further in close association with peptide-rich membrane assemblages, preferentially conducting e^-^ or h^+^ away from the photoactive source. In modern cells, for example, such a task is facilitated by various membrane-embedded terpenoid quinones, in collaboration with protein-bound heme groups and/or FeS clusters. Rudimentary electron transfer chains as such could well have evolved early on, directly feeding part of the photon energy into biochemical redox reactions.

Nowadays such membrane-embedded electron transfer chains result mainly in the build-up of cross-membrane proton gradients, which are subsequently exploited by reversed proton pumps for ATP production. This results in higher yields of utilizable energy. Such intricate rotary pumps, however, together with proton-tight membranes, are quite advanced indeed-only to be added to the biogenic inventory at a later evolutionary stage [83]. Moreover, in the tiny prokaryotic cells that today are the paramount experts in primary energy acquisition, both electron transfer chain and proton-driven turbines to generate ATP are embedded in the all-enclosing cell membrane, together with a host of other components engaged in communication and material exchange with the outer world. Again, this is a highly advanced combination of sophisticated traits, as collected and compacted on a miniaturized scale, not likely to be of early evolutionary origin.

**Figure 4:**
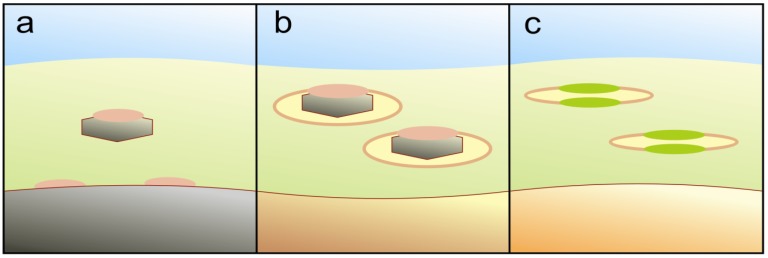
Tentative transition of energized electron transfer from mineral grains to membrane assemblages. (**a**) Peptide-rich micellar patches (pink), together with a rudimentary electron transfer chain, can have assembled at the substratum surface, between the precellular organic matrix (light green) and a larger external photoactive particle (grey), or in close contact with colloidal grains inside. (**b**) Fully enclosing such colloidal particles in membrane-bounded vesicles could render charge separation more effective. (**c**) Incorporating organic pigments (green) in the peptide-rich reaction centers could shift the effective bandwidth into the visible part of the sunlight spectrum.

The communal precellular matrix, as envisaged here, was already bound to make a living on energetically sound principles, but it was likely of a rather diffluent nature with no distinctly definable morphology early on. With energetically unfavorable properties at an extensive and pervious outer boundary in mind (see Section 8.7 for a late appearance of diglyceride lipids), I here consider a stepwise path for the internalization of energy procurement as sketched in Figure 4. (a) Micellar patches of hydrophobic peptides, in cooperation with lipophilic organic redox carriers, could selectively divert at least one partner of a reactive e^-^ / h^+^ pair away from the mineral surface. (b) Full membrane inclusion of internalized colloidal MeS particles offers the possibility of inducing oxidative and reductive processes separately, on either side of the intervening membrane. Also, the biogenic matrix would thereby become independent of coarse graininess in the surroundings. (c) Adding organic pigments to the peptide-dominated reaction centers could progressively utilize the visible part of sunlight and render the photoactive vesicles independent of colloidal minerals. This would effectively extend the biogenic zone to below UV-absorbing protective layers. Later on, it should be considerably easier to consolidate small, specialized internal vesicles as ion- or proton-tight compartments, than conferring such properties to the entire external boundary.

### 4.5. Additional Potential of UV-Facilitated Biochemistry

Besides, the biogenic potential of UV-triggered geochemical effects is not confined to primal photosynthesis alone, although systematic investigations into other supportive reactions have barely just begun. Of older vintage, and little cited since, energy extracted from UV light can be stored in energetic bonds of thioester linkages [84,85] which, in turn, are convertible to acyl-phosphates and to pyrophosphate [85,86,87,88]. Mechanistically, UV absorption by aldehyde moieties was assumed to drive a catalytic thiol-disulfide cycle in the formation of the thioesters [85]. Thus, thioesters and related compounds may have served as the primordial energy source of life [89,90]. Also, the first energy-rich C-O-P-type compounds, catalysed by clay, can have formed under plausible hydrothermal conditions [91,92].

However, while submerged hydrothermal vents attracted much attention, the interest in primal photochemistry subsided for a couple of decades-only to be revived more recently. In addition to primal photosynthesis in terms of carboxylic acids, as mentioned above [5,9], a remarkable photochemical step was discovered in the synthesis of activated pyrimidine ribonucleotides, which happens to bypass free ribose and canonical nucleobases as such, but utilizes arabinose amino-oxazoline and anhydronucleoside intermediates instead [93,94]. In this novel integrative pathway, a common precursor (2-aminooxazole) contributes its atoms to both base and sugar moieties in the final nucleotide products. Yet more instructive is the dual role of phosphate, which acts both as a catalyst and a reactant in this mixed reaction. Also, prolonged UV irradiation in this approach destroyed a plethora of side products so that canonical pyrimidine nucleotides prevailed as ultimate survivors-ribocytidine primarily, from which ribouridine arose by UV-induced conversion. Moreover, consolidating the thioester-to-phosphate relay mentioned above, uracil itself has photo-catalytic activity by facilitating the synthesis of acetyl phosphate from thioacetate by UV light [95], considered to represent a key reaction in protometabolic networks.

This new wave of mixed-system approaches promises a lot to be discovered, as ever more ingredients are subjected to cooperative interactions in carefully controlled experiments. Hitherto, the choice of initial ingredients has largely been motivated by the prevailing paradigm that polymeric RNA alone were the critical factor for bridging the formidable gap from small organic molecules to cellular life. Adding amino acids to such experiments may well bring complementary perspectives to testing.-Will UV irradiation (under anoxic conditions, and in combination with thiols, phosphates and various carbohydrates) be capable of activating amino acids for peptide bond formation in stochastic chain growth?-If so, this would certainly substantiate the hypothesis promoted here that peptides and RNA-like oligomers could have interacted and coevolved from very early beginnings.

## 5. Early Protogenes

In the *photoactive MeS scenario*, primordial organic polymers were strongly selected for UV photostability, giving base-paired RNA-like nucleic acids and structurally consolidated peptides some headstart advantage. Either type of chain molecule is composed of chemically similar-*modular*-subunits, with a virtually unlimited potential of sequence variation. Together, as based on various complementary properties, both chain types engaged in an ever more sophisticated cascade of cooperative coevolution.

Most significantly, base-paired RNA (as well as double-stranded DNA) conceals essentially all the hydrophobic surfaces internally as base stacking along the helical axis, whereas the water-exposed outside is dominated by strongly polar phosphate residues in the backbones. Peptides and proteins, on the other hand, tend to coordinate their backbones by regular intra- or intermolecular hydrogen bonds, exposing their variably polar or hydrophobic side chains for secondary lipophilic interactions-including the potential of membrane attachment or integration.

To start with, the assumption of UV-stability as primal selection factor would favor the emergence of base-paired stretches of double-stranded RNA irrespective of preferential sequences in the individual strands. Initially, that is, complementary synthesis of any sequence would serve the entire system equally well. This would call for the emergence and consolidation of sequence-independent replicating activities and large populations of different molecules, representing more or less stochastic sequences. Many of these would by chance show additional properties, initially of rather low and overlapping specificity, yet slightly advantageous to the overall system. Such systems, therefore, were *highly redundant* in terms of functionally equivalent yet weakly interacting components [96]. From there, self-simplifying and self-complicating trends went hand in hand, leading to fewer yet longer sequences with progressively higher degrees of functional specificity. Such advanced and functionally specific sequences, in turn, could also diverge again and adapt to related or novel roles.

Arguably the greatest mystery in the emergence of life enshrouds the establishment of the *genetic code*, which nowadays is practically universal throughout the entire biosphere. This common framework comprises the mechanism of ribosomal synthesis and the specification of amino acid sequence by the order of particular base triplets in the encoding mRNA, as mediated by dual specificities of different tRNAs for both codons and amino acids, and corresponding dual specificities of the activating aminoacyl-tRNA synthetase proteins.

Much discussion has focused on tentative origins and evolution of the genetic coding rules as such [97,98,99,100,101,102], which is largely beyond the scope of the current review. With rather few exceptions, the canonical genetic code is universally the same across all present forms of life [98]. Also, the overall assignment of codons to the various amino acids appears to be highly optimized [103,104,105]. The most outstanding degrees of order and optimality concern the distribution of hydrophobic potential in the various amino acid side-chains across the canonical codon table. This testifies to the pivotal importance of non-polar peptides (a property distinctly lacking in bona fide nucleic acids) in the modular and coevolutionary self-organization of RNP complexes in primordial biogenesis. Not the least the internalization of electron exchange reactions must have been very much dependent on the formation of hydrophobic pockets between lipophilic peptides.

As seen from a refreshingly novel approach, the overall universality and remarkable optimality of the canonical genetic code itself allows far-reaching inferences about the collective dynamics of early evolution, providing "*strong clues to the nature of early life, and in particular its essential communal character*" [106]. This conclusion underlines the general notion that essentially all prebiotic components were highly miscible at horizontal levels [107], making early evolution inherently reticulate, and genealogical descent in a modern sense was not yet uniquely definable.

The main arguments perceive the evolution of the genetic code as gradually passing from highly ambiguous to uniquely specified codon assigments and decoding mechanisms. For quite some time, therefore, the evolving substratum must be tolerant to considerable ambiguity in generating protein sequences. In terms of game theory, this tolerance to ambiguity is intimately entangled with communal descent and reticulate evolution, which is driven by widespread *innovation sharing* as the superior parameter. It is mainly during this phase of ambiguity and redundancy of multiple components that the collective coding and decoding system is evolvable at all, *i.e*. free to optimize performance and functional efficacy in various dimensions all at once [105,106]. In particular, the speed of approaching such optimality critically depends on the effective pool size of the communal substratum, capable of sharing eventual innovations. In the long run, therefore, the largest innovation-sharing community is fastest in consolidating a universally optimized coding/decoding system, and for that matter alone will be able to outcompete most or all alternative life forms. In other words, it does not pay off at all genetically to withdraw into "splendid isolation" too early. Not having optimized its code beforehand, that is, a small cell-like entity trying to be self-sufficient will inevitably lose out to large communally evolving associations.

Generally speaking, the concepts of *modularity* and *self-organized modularization* are used more distinctly in the related field of "*artificial life*" [108,109,110], than in biology at large, or for that matter, in origins-of-life research. To be sure, the genetic decoding system itself has long been recognized for its modular organization of various components [111,112], but evaluating biological modularity for its evolutionary significance as such is of fairly recent provenance [113,114]. Relatedly, *swarm intelligence*-a technical term originally borrowed from social insect behavior-has rapidly pervaded the fields of adaptive network programming and robotic engineering [115]. Applying this concept to hierarchical (or multilevel) system structures in adaptive interactions, can lead to highly effective optimization algorithms, as compared to single-level simulation [116]. Presumably, similar principles have influenced the adaptive evolution of functionally interconnected macromolecular associations at various subcellular levels, well before the manifestation of propagative cellular identity. As for the still enigmatic emergence of *Darwinian evolution*, the extended concept of *hierarchical clonality* can hopefully help to overcome one of the major controversies about tentative origins of life [2].

- Was cellularization a very early precondition for *Darwinian evolution* as such?
- Or was it rather a relatively late manifestation of *cellular escape*, after a long period of subcellular evolution in indistinctly bounded assemblages of macromolecular hydrogels?

Quite worthy of note is the conclusion that the emergence of novel modularity in organisms evolving in response to changing environments heavily depends on the opportunity of *lateral / horizontal gene transfer* [117], and the evolutionary trajectories recorded in microbial genomes bear ample witness to this notion [118]. Relatedly, the speed of evolution in general depends on recombinational exchange more than on other relevant parameters [119], and recombination is indeed considered a fundamental facility of extreme antiquity [120,121]. Similarly, the evolutionary impact and advantage of *lateral / horizontal gene transfer*, as opposed to *vertical* / *clonal inheritance*, must have been highest at the dawn of cellular life [122].

## 6. From Genes to Chromosomes

Genes are modular entities themselves, composed of variable sequences. Recombining small modular units of emerging functionality in various combinations is the most powerful mode of exploring the vast range of sequence space for local optima at increasing chain length of product [123,124]. Intermolecular exchange events have likely been very important for early genes in the making, as well as their progressive gathering on plasmids or chromosomes. Already when RNA established itself as genetic material, it was critical to distinguish genomic RNA-a primordial *germline* [125]-from processed and catalytically functional ribozymes [126]. Operationally, that is, genetic replication and transcriptional processing had to be kept apart quite early on. As early genes and functional ribozymes were chemically very much alike-consisting of bona fide RNA in either case-differential cues to be discriminated must have developed at the sequence level. For that matter, contemporary ribozymal RNA-protein (RNP) complexes are especially suited to process certain RNAs in various sequence-specific ways, such as 5'-trimming, pseudouridylation, 2'-O-methylation or intron splicing. All of these qualify as ancient relics from a presumptive *RNA world* scenario [127,128]. Intron splicing, in fact, provides an intricate means of genetic recombination at the RNA level, especially insofar as it can occur *in trans* between different parental molecules [129,130].

According to the *introns-first hypothesis* [127,128], the originally ribozyme-encoding genes of the RNA world gave rise to introns later on, when the corresponding spacer regions attained new functions as guiding rails in protoribosome-mediated peptide synthesis. In parallel with the consolidation of the genetic code, these guiding RNAs, in turn, evolved into protein-encoding exons of bona fide mRNAs, and most introns abandoned their primary function. The *mighty-introns model* [126] states about the same, but also adds the notion that the splicing cues in the RNA sequence contributed critically to the operational distinction between + and - strands, containing functional products and complementary sequences, respectively.

From the perspective of early protein evolution, the possibility of exon shuffling appears especially appealing [131,132,133,134]. If small individual exons evolve into protogenes for interactive binding motifs or functionally consolidated protein domains, then occasional recombination events between introns on different RNA molecules would connect the flanking exons-as well as their encoded protein domains-in novel combinations. As stated above, this should provide effective means of sampling vast extents of sequence space for functional utility.

As particular genes became responsible for specific metabolic functions, it also became useful to keep functionally interactive partnerships together over time. There are several biologically relevant modes to provide such physical linkage. Most directly, several genes can be collected on common RNA (or DNA) molecules: plasmids or chromosomes. Also, groups of related genes on different molecules can be anchored at external scaffolds and/or be gathered in closed compartments. The gathering of coding regions for functionally related proteins on a common translatable RNA (as a "*poly-cistronic operon*") may well have started at the *RNA world* stage already. Notably, the clustering of genes for rRNAs and/or ribosomal proteins appears to precede the split into bacterial and archaeal lineages [135,136], as taken up below (Section 8.7). Also, the gathering of entire operons for particular metabolic pathways can have led to the assembly of the first plasmid-like entities [137,138,139].

It has probably taken considerable time of accretional evolution to gather essentially all the genes of any organism's genome on a single chromosomal entity, and only bacteria and archaea (collectively referred to as prokaryotes) follow this pattern today. Also, all free-living cells today have DNA as genomic material, which is inherently more robust and repairable than RNA. Only certain virus genomes still consist of RNA, but are ~100-fold shorter than bacterial or archaeal chromosomes. On the other hand, compounding all the essential genes of a particular organism on a single chromosome is not the only *evolutionarily stable strategy* (ESS) to keep entire genomes closely associated, since no eukaryotic organism ever does it that way. Moreover, while nuclear genomes of eukaryotic cells are distributed over numerous linear chromosomes, prokaryotic plasmids and chromosomes are usually circular. Quite different mechanisms govern genome organization and regular chromosome segregation during the respective cell division cycle in either case [140,141,142]. Which of these regimens is evolutionarily older than the other is still a matter of dispute.

The very early RNA molecules were probably linear chains altogether. Hence the circularity of certain molecules (foremost DNA) is bound to be some secondary derivation later on. To be sure, circularization as such can protect the ends from preferential breakdown, but there are other means of end protection all the same, and eukaryotic telomeres, in particular, appear to be linked down to an ancient RNA world directly [127,143,144]. Arguably the biggest advantage of circular genomic entities is in rolling-circle amplification from unidirectional replication origins [145]. Not inconceivably, circular bacterial chromosomes may have emerged from giant amplifiable plasmids that shifted to bidirectional replication origins and managed to gather all the relevant genes for organizing entire cells on a single genomic entity.

At any rate, the gene-supported precellular systems had to remain robustly self-sustainable during an extended era, when many primordial genes were dispersed over individual molecules or rather short concatenates. The time it took to collect the essential genes of any organism as a readily and faithfully transmissible package-the particular genome-can be referred to as the *pre-genomic era*. It was argued above that life-like ecosystems existed well before the emergence of recognizable-*modular*-organisms. By the same token, early genes can have emerged, cooperated and evolved without being integrated into well defined-*modular*-genomes. To rephrase this notion in the jargon of environmental DNA sampling, *meta-genomes* of entire ecosystems preceded the consolidation of particular organisms with modular genomes for quite some time.

## 7. Cellular Emancipation and Escape

A self-contained and propagative cell is the most prominent modular entity of living matter in the modern biosphere. There are two basic types: tiny prokaryotic bacteria or archaea, apparently quite simple, vs. larger eukaryotic cells, which even in protists show complex internal substructure and often are part of multicellular organisms, such as fungi, plants and animals. Proliferating cells of either kind undergo characteristic *cell division cycles*, thereby securing that equivalent amounts of genomic DNA be transmitted to all the progeny, each time a parental cell divides. Such cycles involve intricate transitory changes in gene and protein activities, which are diligently orchestrated over time.-How could precursory life-like systems remain stable or progressively expand when consolidated genomes or tightly controlled division cycles had not yet been established?

### 7.1. The Coenocytic Alternative to "Cell-like Vesicles Too Early"

Remarkably enough, cell-like non-cellular life forms do in fact exist-collectively referred to as *coenocytes*. These are multinucleated (syncytial) eukaryotic super-cells, ocurring in diverse organisms, such as muscle fibres in our bodies, plant endosperm storage tissue, certain algae, and many non-septate fungal mycelia. Amoeboid, wall-less coenocytes, in particular, have formerly been called *plasmodia*. Now obsolete, this term still reverberates colloquially in *plasmodial slime molds*, such as *Physarum polycephalum*, to describe the entire phylum of myxomycetes or Myxogastria [146,147]. Conceivably, an amoeboid proto-coenocytic model [4,148] is best suited for dealing with stochastic fluctuations of gene number and specificity in the *pre-genomic era*.

This model combines the panmictic properties of Woese's *progenote* concept [106,107,149], as well as the sampling aspect of Kandler's *pre-cell theory* [150,151,152,153], with internal membrane diversification early on [75,154,155]. The high miscibility initially required could not prevail without means of large-scale confluence, but active protocytoplasmic movement did not exist to start with. Some forced environmental mixing could have made up for the lack of mobility in the protobiotic organic matrix itself. Thus, similar to modern syncytia of plasmodial slime molds, which fuse quite freely upon chance encounters of like genotypes [156], the sessile proto-coenocytic plaques of the pre-genomic era may readily have fused as well, so as to reconstitute contiguous layers around the silty sedimentary particles, whenever the shallow biogenic banks resettled after being reshuffled by turbulent periodic water currents. Flexible and fusible outer membranes may thus be considered a primordial trait.

### 7.2. The "Karyogenic Proto-Coenocyte Hypothesis"-Emergence of Multiple Protonuclei

In the unseptated deposits of organic hydrogels, chromatin-like complexes of nucleic acids tended to phase separate as gene-rich blobs, which in turn could interact with flattened internal membrane systems. Such *visco-elastic phase separation* occurs both in bacterial nucleoids [157] and in eukaryotic nuclei [158,159]. This is one way of rationalizing the tentative emergence of protonuclei [75,160]-the *Karyogenic Hypothesis*. This concept resembles the regularly recurring regeneration of the nuclear envelope in *open mitosis* of eukaryotic plants and animals [161], as opposed to *closed mitosis* in fungi and many protists where the nuclear envelope remains intact throughout the entire cell division cycle. By and large, the *Karyogenic Hypothesis* appears to represent a more facile model to rationalize the primordial emergence of genome-enclosing nuclear envelopes [162], than the currently prevalent *Endokaryotic Hypothesis*, viewing nuclei as tentative relics from endosymbiotic origins in fusing two full-fledged prokaryotic cells [163]. I herein propose its extension as *"Karyogenic Proto-Coenocyte Hypothesis".*

Carl Woese envisioned "*progenotes*" as highly multi-genic, yet anucleate, protocells early on [107]: "*The structure of these genomes must reflect the primitive evolutionary dynamic in general. Therefore, I see the progenote genome as organized rather like the macronucleus of some ciliates today: it comprised many small linear chromosomes (mini-chromosomes), each present in multiple copies. Each chromosome was ‘operonally’ organized, that is, functionally or structurally related genes were grouped together. ... Cell division occurred in the simplest way possible, by a physical pinching of the cell into two approximately equal halves.*"-Quite similar considerations apply to the protonuclei envisioned here, together with certain *bonus qualities* inherent in wide-spread sharing of a common cytoplasma-like matrix. Different from modern nuclei, however, the emerging protonuclear compartments in a common matrix would not yet have established any reliable partition mechanism, leading to highly heterogeneous distributions in terms of gene content to start with. As demonstrated by the multigenomic population structure of coenocytic arbuscular mycorrhizal fungi (Glomeromycota) [164,165], unusually large amounts of polymorphism and long-term nuclear heterogeneity are not detrimental as such to long-lasting evolutionary success.

The main advantage of multiple protonuclei being embedded in large expanses of shared resources in a common cytoplasm is in mutual cross-feeding of complementary activities between many protonuclear entities, which individually are more or less suboptimal or partly deficient. Such individual deficiencies inherently arise from stochastic sampling fluctuations in the initially inaccurate and mostly unequal "pinching" process. It is the buffering capacity of extensive and communally shared cytoplasm that gives a plethora of early protogenes and rather fuzzy propagation mechanisms the evolutionary time required to optimize their interaction networks, as well as to establish proper means for faithful segregation at each division.

The inherent benefits of protonuclear compartmentalization become more evident by a conceptual comparison with corresponding drawbacks of haphazard protocellular division at a precocious stage. For as long as many (mostly linear) genomic fragments are not yet consolidated on a common chromosomal genome, a stochastic and mostly unequal 'pinching' process would lead to a vast majority of deficient cells. A similar process applied to protonuclear compartments, however, would not kill the common coenocytic matrix, since functional complementation would integrate over a large population of individually deficient protonuclei. In such a scenario of *hierarchical clonality*, *Darwinian evolution* can have commenced rather gently-acting for quite some time on protonuclear subcellular compartments in a common cytoplasm, rather than on genuine, clonally diverging cells. Also, the same principles of communal optimization that have been implemented in the perfection of ribosomal protein synthesis [105,106] can have guided and optimized the mechanisms of mitotic nuclear division well before this capacity became coupled to regulated cell division.

### 7.3. Cellular Escape Events

As optimization progresses, the system reaches some point of diminishing returns, and the buffering constraints of the communal matrix will dwindle. Thereafter, more or less autonomous subsystems may successfully commence evolving on their own. This marks the emergence of vertical inheritance at the cellular/organismal level. Operationally speaking, such a transition should be accompanied by the liberation, release or escape of cellular entities from some precursory system, which must have prevailed without being cellularly organized beforehand. The catch-word *cellular escape* has been coined for this process [166], albeit in the particular context of a very specific model, implying *mineral-bubble* compartments at submerged hydrothermal vents as the precursory stage [19]. Although this iconic model is currently prevalent in various forms [20,21,22,83,167], it does not readily provide for long-range macromolecular miscibility between such stationary-and virtually stagnant-mineral encrustations. Accordingly, I should like to retain *cellular escape* as a general term for such a pivotal transition, and rather reapply it in a somewhat different context [4], to wit, a photo-driven ecosystem in the upper-layer *porespace* of *shifting sedimentary banks*, as described above.

As system-supportive innovation rates taper off, the vast buffering potential of communal sharing becomes a burden rather than an unmixed blessing. This is because the buffering does not only maximize positive input by pooling resources over larger volumes, it also tends to accumulate unproductive ‘dead weight’ over time. Hence, internal pressure rises to concentrate the most productive traits in modular self-contained units, provided these entities remain capable of self-renewal and self-similar propagation in the long run.

The range of such attempts to gain some modular genetic autonomy is large. It spans from sheer selfishness of infective viruses [22], over transmissible and amplifiable plasmids carrying metabolic cross-feeding potential, as modeled by the plasmid-borne pathway of nitrogen fixation in rhizobial bacteria [168], to full-fledged bacteria-like cells [166]. Conceivably, such newly emerging bacteria-like cells obtained their circular unified chromosomes from preexisting circular plasmids that eventually had accrued all the household functions needed to maintain cellular autonomy on a single loop of DNA [169]. In the sunlight-exposed scenario favored here, and as potential profit margins are highest at the foremost frontier of primary production, the first genuine cells likely arose as specialists in photoactivated CO_2 _fixation. For quite some time, however, the earliest cells *escaping* in such a way from their communal source had to coexist with the progenerative matrix at large. Multiple events of *cellular escape* can have occurred repeatedly and independently (Figure 1), each time carrying with them different samples of genetic elements, as drawn from the considerably larger gene pool remaining in the communal matrix, in accord with Kandler's *pre-cell theory* [150,151,152].

## 8. Rethinking the Primal Dichotomy and the Primordial Trefoil in the Universal *Tree of Life*

### 8.1. Nesting Eukaryotes Between Two Phylodomains of Prokaryotic Lineages

The basic assumption of the concepts developed above is a complex communal system at the common source or root of the major lineages toward current life, which subsequently diverged into ever more specialized organisms. The proto-organismal system at the enigmatic root goes by the acronym of LUCAS-here read as the *Last Universal Communal Ancestor State* (see ref. [4] for alternative readings, and references on precursory terms as well). At this primordial state, the communal system was not yet dispersed over many individualizable lineages of organisms, but it must already have comprised all the activities required for a self-sufficient ecosystem to be maintained autonomously.

There is a trefoil of three major branches that has to be connected to the LUCAS in a reasonable way-the *phylodomains* or *superkingdoms* of current life. As two of these comprise different kinds of prokaryotic cells-here referred to as the *primal dichotomy* into **Bacteria** and **Archaea**-this sets **Eucaria** apart as the enigmatic third brick in a conceptual puzzle. As Eucaria and Archaea are often recognized as sister clades in deep-reaching phylogeny (see below), the *primal dichotomy* is assumed to separate bacterial and archaeal/protoeukaryotic stemlines early on (Figure 1).

It is of major concerns that all the eukaryotic cells are so radically diverse from both kinds of prokaryotic counterparts that hardly any obvious theory a posteriori, by smoothly extrapolating from modern organisms to the distant past, can readily rationalize their common origins from a single source. The grand schism between the prokaryotic and eukaryotic ways of life is not merely confined to the internal organization and complexity of the respective cells, it also marks profound differences in evolutionary mechanisms and kinetics across the prokaryote-eukaryote divide [170]. Conceptually speaking, the eukaryotic way of life appears more easily derivable from a complex communal source than either one of the prokaryotic domains. Yet this runs counter to the often repeated mantra that bacteria-like prokaryotic cells must have preceded protoeukaryotes.

A comprehensive comparison of evolutionary principles in eukaryotic and prokaryotic organisms, as well as their respective cellular organization, is presented by Cavalier-Smith [171]. Still, though, the author does not question the basic *prokaryotes-before-eukaryotes* paradigm, but rather attributes acquisition of the full range of eukaryotic innovations to an unprecedented burst of vastly accelerated "*microevolution*" from fully established bacteria-type cells. Arguably-from the sketch of evolutionary perspectives promoted here-his earlier attempts to popularize Blobel's inverted vesicles [154] as "*obscells*" [155] appears closer to the elusive succession of historical events. As brought up repeatedly in the current paper, I do not share much trust in the explanatory power of miraculous events or episodes. Instead, the fundamental insight that the falling into place of multiple interactive components can be highly facilitated in a large communal matrix system, as exemplified by the emergence and optimization of RNA-encoded protein synthesis [106], should likewise be considered and critically evaluted for primal eukaryogenesis.

### 8.2. Can Fossil Scarcity Constrain Eukaryogenic Time Scales?

When exactly did eukaryote ancestors first appear on early Earth?-As of yet, this pivotal question cannot conclusively be answered from fossilized remains alone. In the scenario developed here, it can hardly be expected that naked amoeboid protoeukaryotes should leave any recognizable fossilized traces at all. For prokaryotes, too, the early fossil record is very scanty and spurious indeed, essentially being limited to filamentous cyanobacteria-like threads encrusted in ancient stromatolite remains [172]. Thus, a long-lasting controversy still waits to be resolved between proponents of early and late protoeukaryote appearance [4,148,169,173,174,175] (for tabulated comparisons of various evolutionary models for eukaryote origins see [174,176]). Unfortunately, though, adherents to either position tend to argue in partly incommensurable terms. At the heart of this dispute, the contending parties profoundly disagree on the validity of certain axiomatic assertions, as to the relative importance-and shifting impact over time-of widely disparate mechanisms in evolutionary innovation. For the somewhat bewildered onlooker to this debate, the gist of it boils down to the following confrontation: "*Eukaryotes arose from prokaryotes*"-period! [177], *vs.* No miracles, please, nor "*mechanisms founded in unfettered imagination*", at later and well-consolidated stages of biotic evolution! [178]. I find taking the latter stance far more appropriate.

Dagan *et al.* [177] still heavily rely on the apparent absence of "*diversified and unequivocally eukaryotic cells*" in the documented fossilized record before ~1.5 Ga of age for drawing their conclusion that "*eukaryotes appear about 2 billion years later in the geological record than do prokaryotes*". Yet, the shakiness of such spurious *absence of evidence* (which is not equivalent to *evidence of absence*) is underlined by the recent presentation of numerous globular microfossils, ~3.2 Ga of age, which are surprisingly big (50-300 μm) [179]. Perhaps they resembled eukaryotes already, or certain prokaryotes of that era were substantially larger than modern bacteria. At about the same time of genetic innovation, the *Archaean Expansion* of bacterial lineages was accompanied by a formidable burst of new gene families, as closely followed by a similar counter-spike of gene loss events in major branches [180].

As cellular complexity, thus, was already considerably larger around the base of bacterial radiation than hitherto appreciated, the internal membranes prevailing in the somewhat neglected *planctobacterial* superphylum are cast into a new light [181,182]. Even though planctobacteria as such may not be considered to represent the elusive missing link toward eukaryogenesis directly [183], their common descendence from a communal source is not entirely out of the question [184].

### 8.3. Ambiguous Leads to the Emergence of Three Phylodomains

When the conceptional split of prokaryotes into Bacteria and Archaea was first discovered from systematic rDNA sequence comparisons [107,149,185], it came very much as a surprise. On this nascent bifurcating tree of rDNA relationship, Eucaria were found to be more closely related to Archaea than to Bacteria. When protein squences were analysed accordingly, however, the canonical pattern of a uniquely bifurcating tree became exceedingly blurred or '*fuzzy*' at the edges. To wit, not all the corresponding genes happened to follow identical trajectories. For that matter, a universally valid *tree-like* phylogeny only holds up for a modular, relatively narrow core set of protein-coding genes, most of which have functionally coevolved with protein translation [186,187,188].

The ambiguity outside the modular core set is most pronounced within both prokaryotic phylodomains, where it has been ascribed to phylogenetically high rates of *lateral / horizontal gene transfer* [189,190,191], especially in the earliest phases of bacterial diversification. Referring to this wide-spread reticulate pattern of phylogenetic relationship in prokaryotes, the iconical *tree of life* metaphor embodies a rather *fuzzy tree*, at best, and might as well be rephrased as a *rhizome of life* [192,193], for which the full extent of all its ramifications still remains to be charted.

In modern eukaryotes, however, the phylogenetic impact of *lateral / horizontal gene transfer* is much less apparent, at least between unrelated species, although acquisition of alien prokaryotic genes in fungi is well recognized [194]. Of all eukaryotes, for that matter, fungi have most effectively engaged in direct competition with bacteria as to recycling of organic matter, even though they have lost the capability of phagocytosis. Besides, the ancient endosymbioses of mitochondria and plastids have led to massive influx of prokaryotic genes into eukaryotic nuclear genomes already in the distant past [195,196]. On the other hand, reticulate inheritance as such-within a particular species-is firmly institutionalized in eukaryotic inheritance, since facultative or obligatory conspecific sex is an integral part of most eukaryote life cycle strategies, which essentially goes back to before the last eukaryote common ancestor (LECA) [148,197,198,199]. A universal precondition for eukaryotes to engage in sexual reproduction is their readiness to engage in conspecific cytoplasmic fusion, so as to merge entire cells and genomes into novel integral entities. This peculiar communal property is entirely lacking in the prokaryotic world.

### 8.4. Ecological Considerations-Engulfment and Gamete Fusion

Some ecological considerations, too, should be appropriate to a coherent narrative as a prospective goal. The categorical difference between modern eukaryotes and their prokaryotic counterparts does not only amount to intracellular complexity. It also concerns different roles in overarching ecosystems, as well as different modes of selective evolutionary change. This divergence concerns a functional split between primary production on the one hand and predatory feeding on the other. This is mutually exclusive in the sense that all primary production of new biomass is the sole responsibility of prokaryotic cells (or organellar symbionts of prokaryotic descent), while the engulfment of particulate food items, other living cells included, is solely accomplished by eukaryotic cells. Such functional dichotomy is likely of very ancient origins as well. This is reflected in a bold, judicious and integrative statement, "*it is inappropriate to regard the prokaryotes as primitive and the eukaryotes as advanced-it is more fruitful to view the two groups as having adopted successful but alternative evolutionary strategies, in which prokaryotes have exploited the advantages of miniaturization and eukaryotes those of size*" [200]. Similar arguments reverberate in Forterre's *Thermoreduction Hypothesis* [173] (see below).

Phagocytosis by engulfment is indeed a very ancient trait of eukaryotes in general [171,201,202], which most likely has taken a long time to evolve in the protoeukaryotic stemline [203,204]. Also, extended evolutionary periods without any feeding predators are quite unlikely [205]. The recognition of endocytosis-like protein uptake in planctobacteria [206] is compatible with the notion that such endomembrane-facilitated uptake preceded the *primal dichotomy* of the phylogenetic *tree of life*, where protobacterial ancestors split away from the still common stemline of archaeal-protoeukaryotic ancestors, although genuine homology between these uptake systems has been questioned [183]. In the scenario favored here, the operational prerequisites for phagocytosis are partly overlapping with physically similar preconditions for sexual gamete fusion, and both complex features may naturally link back to a primally cell wall-less communal state. Alternatively, the operational origins of phagocytosis alone [207], or together with sexuality as related synergic features [208], have also been discussed in the context of biofilm-embedded models of eukaryogenesis from symbiotic mergers. Yet, these models leave too many unrelated complex eukaryotic core traits unexplained (see below) to be considered as a comprehensive theory.

Seen from the vantage point of a coherent and communal precellular molecular ecosystem, as discussed above in preceding sections, a direct path to eukaryotic cell organization poses no mystifying conundrum at any step. Conversely, quite serious objections can be raised on various issues about a tentative prokaryote-to-eukaryote transition at any later and more consolidated evolutionary stage [204,205,209,210,211]. Advocates of *prokaryotes-alone-at-start* models tend to neglect or underestimate the pitfalls and idiosyncrasies of their assumptions [160,163,174,212], and molecular phylogenomics cannot yet discriminate between the opposing views [183,213].

Also of ecological concerns is the presumptive 'ambiente', or environmental setting, around the LUCAS precellular community-say the ambient temperature at life's beginning. Was it hot, or was it rather temperate, on average? In fact, a *hot-start scenario* of primordial biogenesis became fashionable when it was recognized that the deepest branching lineages of both bacteria and archaea comprise of thermophilic organisms [214,215], and proponents of the related deep-sea hydrothermal-vent scenario, in particular, welcomed and embraced this view [19,20,21]. On the other hand, an RNA-based system of early life would hardly work out at thermophilic temperatures [216,217], and the inferred history of temperature-correlated base pair substitutions in ribosomal RNA is indeed incompatible with a common root at thermophilic temperatures [218]. Even at the level of more stable DNA, the thermophilic traits require complex specializations, which most likely derived from mesophilic precursors [219,220]-presumably as convergent innovations and/or lateral gene transfer at the root of either prokaryotic phylodomain. Together with genomic streamlining on a massive scale in prokaryotic cells, this *Thermoreduction Hypothesis* allows protoeukaryotic ancestors to arise under moderate, or mesophilic, conditions, so that they could retain a higher share of primordial remnants from a preceding RNA-dominated era [173], before the thermophilic archaea emerged as well. Notably, the separate emergence of thermophilic adaptations of bacterial and archaeal lineages in parallel has also been inferred from comparative proteomic analyses [221].

### 8.5. Continuity of Ancient RNA Functions

Last, not least, the presumably very ancient origins of the protoeukaryotic stemline are reflected in a plethora of functional RNA molecules. Assuming *continuity of function* in complex RNA-based traits as a potent selective principle, this tentatively links eukaryotes directly to a veritable RNA world when coded protein synthesis was just emerging [127,128,169,203,210,222,223,224,225,226,227]. Among these functional RNAs or RNP complexes, the formidable spliceosomes are considered of particular importance.

Nuclear spliceosomal introns and organellar group II self-splicing introns are certainly related [228,229], but their origin is not well understood [230]. It is merely hypothetical that the multipartite spliceosomal machinery might have originated from compact self-splicing elements [228], and that its emergence were triggered by a chain reaction after the acquisition of bacterial proto-mitochondrial endosymbionts in an archaeal host [212]. More likely, though, the self-splicing transposable elements have in turn derived from preexisting multipartite components. Moreover, the massive intron expansion in the postulated chimeric fusant clone as such is exceedingly improbable [211]. Even more far-fetched are the correlated assumptions of inventing both spliceosomes and nuclear envelopes ad hoc in the same fusant clone, so as to limitate intron expansion and to separate splicing from translation [212].

It is reasonable enough to presume a precellular *genetic melting pot* early on, from which the first genomic associations can have coalesced [107], which requires a quite large promiscuous population or molecular ecosystem to be robustly self-supportive and able to optimize the basic prerequisites for cellular autonomy [106]. But starting a secondary genomic meltdown from a single cell-the first host cell of the intron-bearing endosymbiont in an archaeal host [212]-appears alarmingly self-destructive. Proposing to relieve the threat of intron spread, in a fully established protein-synthesizing organism, by ad hoc invention of the largest RNP complexes ever formed must be one of those "*mechanisms founded in unfettered imagination*" [178]. Rather, the composite eukaryotic spliceosomes bear all the hallmarks of originating from the same type of precellular (*proto-coenocytic*) genetic melting pot that also gave rise to ribosomes and other RNP machines [128].

### 8.6. Significance of a Complex Protoeukaryotic Stemline

It is widely recognized by now that the *last eukaryote common ancestor* (LECA) was highly evolved genetically [183,202], combining a modular and interactive core set of eukaryote-specific proteins, maintaining a sophisticated cytoplasmic infrastructure and cytoskeleton organization. This begs paramount questions of how long it took to reach that highly interactive stage and of what happened to all the side shoots to be expected in addition to the solely surviving stemline before the LECA [203]. Eukaryote-late models resort to an intense period entailing "*a dramatic acceleration of evolution*" [183], which would be extended and relaxed quite naturally in eukaryote-early scenarios. The preferable *eukaryote-early* alternative, in turn, assumes a complex collective genome for the LUCAS stage already, from which only the prokaryotic descendents lost many traits, owing to severely *r*-selected genomic streamlining. The protoeukaryotic stemline-still lacking mitochondria or plasmids as secondarily acquired endosymbionts-not only maintained the high level of primordial complexity, but could further complexify at a modest rate. A complex (eukaryote-like) collective proteome at the common ancestral root is indeed already emanating from studying protein fold phylogenies, together with reductive tendencies in the prokaryotic domains [231,232].

As for the flexible membranes here assumed to be a primordial trait of the communal stemlines, a recent finding appears quite relevant. Phylogenomic analyses of membrane remodeling have documented that the common ancestors of protoeukaryotes and archaea were considerably more complex in this regard than any archaeal lineage individually [233]. Notably, eukaryotes possess three different systems of membrane remodeling, including vesicle formation, which all can be traced back to the last common archaeal ancestor as well, but different components were apparently lost in various archaeal lineages later on. Together with related comparisons of other cytoskeleton components [234], this indicates that archaea and protoeukaryotes shared a common ancestral lineage of elaborate internal complexity, from which only the archaeal descendents diverged by losing many components in *r*-selected, *reductive evolution*. The molecular signatures of ribosomal-protein evolution, too, appear to underline this point [235].

On the other hand, the apparent crown group radiation from a complex LECA happened rather late, somewhere in the proterozoic era. One way of reconciling this with a longer time of as yet elusive stemline evolution is the potential occurrence of severe bottlenecks, affecting protoeukaryotes preferentially [236]. Such a critical constriction point can have come with the first and most severe occurrence of a global ice age ~2.4 Ga ago, the paleoproterozoic *snowball Earth* glaciation [237,238]. Quite naturally, for that matter, a sparse predatory population of (still unicellular or coenocytic) protoeukaryotic grazers would be most vulnerable, and was driven closest to extinction when their prey of prokaryotic cells severely declined in abundance for an extended period.

The lucky few to survive this ecological collapse have supposedly carried the basic eukaryotic characteristics in combination: cycling between an amoeboid trophic stage and ciliated propagation on the one hand, and between mitotic and meiotic nuclear divisions on the other, as sexually complemented by gamete fusion somewhere in the life cycle, perhaps with cyst-like resting stages as well, and carrying mitochondrial endosymbionts already. The aerobic amoebozoan zooflagellate *Phalansterium* has been suggested as a modeling example [201]. As of yet, it is indirect evidence from geochemical traces alone that links eukaryote-type membrane lipids to the Archean-Paleozoic era: by detecting C27-C29 steranes as tentative sterol degradation products in fossilized organic matter ~2.7 Ga of age [239,240].

As mentioned before, numerous scholars of deep phylogeny have emphasized the pervasive influence of *lateral/horizontal gene transfer* on early prokaryote evolution. This terminology is used as if the classical means of genetic exchange between bacterial lineages-transforming DNA uptake, plasmid transfer, and prophage transduction-were solely present in the communal era of common ancestors as well. This need not necessarily hold true and is more likely not the case. Alternatively, the promiscuous nature of the communal state of high genetic redundancy can have been based on indiscriminate cytoplasmic fusion early on. From such a complex and highly redundant source to start with, incongruent trajectories of many different genes can subsequently have resulted from lineage-specific losses and selective retention of particular paralogs [241,242,243,244].

### 8.7. Multiple Escape Events at the Base of Prokaryotic Lineages

At some point in early evolution, this communal system split up into two separate branches, which temporarily ceased to exchange genetic information across this *primal dichotomy*, which gave rise to all the bacterial lineages on the one hand, and the archaeal/eukaryotic lineages on the other. The most effective barrier against convective gene flow is physical or geographic separation [245], which should preferentially be considered at this early stage as well [150]. If this deepest split developed already at the stage of sessile precellular systems, the physical distance need not have been large to be effective. Bacterial lineages may have evolved from the bulk of the precellular ecosystem early on, remaining under the influence of sunlight exposure and making photosynthesis more and more effective. A divergent subsystem, however, was displaced into the shade of deeper sediments or muddy waters, where this lineage specialized on heterotrophic recycling of organic matter and lost the potential of primordial photosynthesis. From there the archaea may have diverted in two waves; the extremophilic Euryarchaeota diverged to habitats where protoeukaryotes could not follow, whereas the Crenarchaeota (eocytes) and eukaryotes developed from the remaining lineage. The latter view would offer an alternative to the *eocyte hypothesis*, which has been proposed to rationalize a relatively close relationship of eukaryotes to Crenarchaeota [246,247].

This interpretation of the primal evolutionary branching point implies a precellular system of both sophisticated and frail characteristics. Its most sophisticated legacy is the canonical system of gene-encoded protein synthesis, which all three phylodomains have essentially in common. A rather frail or ill-defined phylogenic signal concerns the putative nature of membrane-like boundaries at the early evolutionary stage of LUCAS. On the one hand, quite sophisticated membrane-spanning proteins can be traced back from all three phylodomains to a common LUCAS stage [248,249,250]. On the other hand, the lipid components in modern archaea and bacteria are different to such degrees that the putative lipid content of primordial membranes is very much in doubt [251,252]. The archaeal isoprenoid chains are ether-linked to glycerol, whereas the fatty acid moieties of bacterial lipids are linked by ester bonds. Moreover, the central glycerol is sterically different, being derived from sn-glycerol-1-phosphate and sn-glycerol-3-phosphate in archaeal and bacterial lipids, respectively.

One way to rationalize this enigma could be that the lipid fraction of the LUCAS primarily consisted of monoglycerides or other single-chain derivatives. Notably, the common precursor of the stereo-isomeric glycerolphosphates mentioned above is the achiral dihydroxyacetonephosphate, from which different dehydrogenase enzymes generate the bacterial- and archaeal-type glycerolphosphates as enantiomeric lipid precursors [253,254]. Hence, dihydroxyacetonephosphate could have been used directly for lipid biosynthesis, yet only accommodating a single hydrophobic chain. In general, single-chain lipids, such as dodecyl phosphocholine or lysolecithins, are detrimental to bilayered lipid membrane structure [255]. In peptide-dominated membrane-like patches, however, they may well be suitable as space-filling units, as experimentally utilized in micelle to 2D-crystal transition of integral membrane proteins [256]. This unorthodox interpretation [4] is compatible with the assumption of a complex LUCAS with locally different membrane assemblies, comprising relatively small peptide-rich patches or vesicles for energetic purposes (Figure 4) and a more fragile outer boundary, which is not yet refractory to promiscuous fusion.

On the other hand, the putative presence in the LUCAS of at least two enzymes engaged in the processing of UDP-N-acetylglucosamine [248] may indicate some potential of cell wall-like glycosylation events outside of external membranes quite early on. This does not mean, however, that rigid cell walls must have been present all the time. The temporary and inducible involvement of UDP-N-acetylglucosamine pyrophosphorylase in cyst wall formation [257], for example, may point in a different direction.

As discussed above (Section 7), the complex and communal LUCAS system has eventually given rise to independently propagating cells in multiple rounds of *cellular escape* [22,166]. Not inconceivably, for that matter, the liberation of propagative modules from a communal matrix may have begun with the periodic encapsulation (*encystment*) of resting stages sufficiently large to regenerate the entire communal system at new locations and/or after periods of harsh conditions unfavorable for continual growth [4]. Such encysted propagules, in turn, could progressively divert to repetitive and controlled cell division, instead of returning to communal regimes of syncytial growth. This sequence of events, in fact, is not as fanciful or outlandish as it may seem to the uninitiated reader. In biological evolution, similar events have actually occurred more than once, as illustrated by the independent regression of spores or gametes to unicellular, yeast-like lineages from different filamentous and multi-nucleate fungi [258,259]. It is also of note that eukaryotic yeast cells mimic prokaryotic cells in their propensity of being subject to *r*-selection during vegetative propagation (maximizing *growth rate* in times of affluence), which favors miniaturization and genomic streamlining [200,260,261,262,263]. Their ability to resort to sexual propagation and spore formation, however, results from an alternative regime of *K*-selection (maximizing *carrying capacity*, and minimizing accidental extinction under adverse conditions).

From some of the first successful *micro-cellular escape* events, major lineages of bacterial and archaeal cells emerged on either side of the primal dichotomy and progressively perfected their streamlined *prokaryotic* way of being. As dehydration resistant traits of dormant cysts or spores often go hand in hand with some degree of heat resistance, such resting stages could not only constitute starting blocks for *cellular escape* events as such, but also serve as *preadaptations* for prokaryote growth and propagation at higher temperatures, in accordance with the *Thermoreduction Hypothesis* mentioned above (Section 8.4).

Any ambitious model to interpret the cladistic relationship between the three canonical phylodomains must face the serious problem of "*how to polarize shared archaea/eukarya or archaea/bacteria characters*" [264]. This also concerns the *Karyogenic Proto-Coenocyte Hypothesis* promoted here, which interprets the emergence of bacterial and archaeal cells as independent *escape events* from a communal, super-cellular, more eukaryote-like matrix.

- How can there be room for *shared archaeal/bacterial characters* that are not automatically represented in eukaryotes as well?

As briefly mentioned above (Section 6), ribosomal protein genes of prokaryotes are linked in functional clusters [135,136], which evidently precede the *primal dichotomy* where ancestors to bacterial and archaeal lineages have split apart. Together with various other highly conserved genes, the ribosomal protein genes form *super-operons* of >20 genes [265], comprising the most conserved assemblies in prokaryotic genomes. This contrasts with eukaryotes, where such genes are invariably and indiscriminately spread out among all the other genes on numerous linear chromosomes.

Before the propagative fixation of entire genomes in individually encapsulated cells, however, the mixed kinetics in partly compartmentalized, yet higly miscible communal super-cells should allow for different means of genomic consolidation to proceed side by side. In the context of the dendrogram depicted in Figure 1, the *ribosomal super-operons* have formed before the *primal dichotomy*, whereafter they diverged genetically into bacterial and archaeal/protoeukaryotic varieties. The latter were still intact at the time of *cellular escape* of archaeal cell lines, when full ribosome-encoding circular plasmids had accrued a sufficient number of additional genes, so as to organize entirely self-sufficient cells for independent propagation. Equivalent processes let bacterial cell lines *escape* on the other side of the *primal dichotomy*.

In terms of internal compartmentalization, it is not inconceivable that differently organized compartments coexisted inside the communal supercells. Multiple linear chromosomes may thus have prevailed in *proto-nuclei*, whereas circular plasmid-like chromosomes settled in a different type of compartment. The latter kind would initially carry most genes involved in protein synthesis and related functions, thus gathering many *proto-nucleolar* activities. Before the advent and perfection of reliable bipartition mechanisms, moderate rates of gene flow between compartments can have resulted in considerable variation in gene number per compartment of either kind. It is from the plasmid-derived category that bacterial and/or archaeal cells can successfully have "escaped" as independently viable and propagatable prokaryotic "micro-cells".

### 8.8. Eukaryotes, Organelles and Megaviruses

In addition to bacteria-type micro-cells, the residual communal matrix also gave rise to larger, more complex, and more sluggishly evolving, yet less numerous *macro-cells* of protoeukaryotic organization, perfecting mitotic nuclear division with multiple linear chromosomes as their common mechanism of a functional cell division cycle. In these, the ribosomal super-operons and other clusters of functionally related genes were evolutionarily scrambled into stochastic order. This was likely accelerated after the consolidation of sexual propagation once per life cycle, where abundantly scheduled homologous recombination became institutionalized in eukaryotic organisms. It is probably a matter of historical contingency (such as an exceptionally severe bottleneck about half way down into life's existence) that no complex organisms related to bacteria left any record, but the only surviving lineage of that kind originated from the "*archaeal*" side of the *primal dichotomy* (Figure 1). Some of these complex survivers specialized in feeding on the more abundant prokaryotic cells. Eventually the acquisition and internalization of *mitochondrial endosymbionts* led to the only representatives of that branch, now having fully established the organelle-assisted *eukaryotic* way of life. To be sure, the impact and importance of such endosymbionts for all the modern eukaryotes is undeniable, and is not questioned in the current paper either. The open question still concerns the putative nature of the host lineage that successfully acquired the first decidedly prokaryotic endosymbiont. Historically, numerous models have been suggested for such a stem line, as comprehensively reviewed in ref. [266]; despite its deceptive title, however, that compilation deals more with origins of endosymbiotic organelles than with origins of eukaryotes as such, or rather the origins of the receiving host line, for that matter.

Besides the emergence of genuine cells, also viral lineages are assumed to have sprung from the primordially communal matrix in different kinds of escape events, devoid of ribosomes [22]. Strong arguments were even put forth to suggest that the crucial transition from RNA to DNA was primarily spurred by viral specialization [267,268]. For most viruses, however, it is virtually impossible to pinpoint their emergence on the canonical *tree of life* by convential methods of genomic alignment, due to their paucity of core genes in common with genuine cells-with a particularly striking exception. In fact, a special group of *Giant Viruses* (*Mimiviridae*, *Poxviridae*, and several others) has been characterized as carrying megabase genomes, including a large variety of core genes likewise found in organisms of all three cellular phylodomains [269,270]. Only ribosomes and energy conversion are completely lacking. These large viruses exclusively depend on eukaryotic host cells. They form a monophyletic group and cluster outside the lineage of extant eukaryotes [271]-thus forming a distinct *4th domain* on a *genomic tree of life*, from which the otherwise implicit notion of cellular autonomy has been removed.

It makes good sense to assume that *Giant Viruses*, too, emerged from the primordially communal matrix itself or closely to it [22], but only on the condition that the same assumption holds for the protoeukaryotic stemline as well. Otherwise the continuity of suitable hosts would be broken, and the alternative assumption of positing tentative prokaryotic host cells for about half the evolutionary time of mimi/pox-type viruses' presumed existence would merely add another oddity to "*mechanisms founded in unfettered imagination*", solely construed to save the elusive postulate that bacteria-like prokaryotes had to precede protoeukaryotes by eons [178].

## 9. Concluding Prospects

"*However disagreeable uncertainty may seem, proclaiming certainty is quite absurd*" [272].

Hypotheses about the historical origins of life as we know it here on Earth are out of reach for experimental verification-or falsification, for that matter. Strictly speaking, therefore, such speculations go beyond the scope of science and technology. This does not mean, however, that science has no word to say about the credibility of any claim to life's tentative emergence. Above all, it is a significant feature in scientific reasoning to call in question stereotyped assertions and reassess their merits in the light of circumstantial information of current scientific standards.

Others have tried before me to debunk the traditional myth that bacteria-like cells as such are less evolved (more 'primitive') than any eukaryotic protists, and that early eukaryotes, therefore, must be derived from preexisting (bacteria-like) prokaryotic cells. Yet, "*Prokaryotes are not just primitive organisms that failed to become eukaryotes. They are very sophisticated in their RNA processing and have an elegant simplicity that is ideal for their lifestyle*" [169]. At any rate, "*it is not known to what extent the 'simplicity' of prokaryotes is due to their 'primitive characters' or to the elimination of ancient more complex characters by reductive evolution*" [273]. It is clearer, though, that "*many stages of evolution appear to have been accompanied by physical loss of superfluous DNA. It is postulated that the genomes of prokaryotes-where almost every gene is represented by one copy only-represent the results of this process carried to its extreme*" [274].

Herein I have added to these notions that the communal nature implied for a large precellular matrix [106,107] more likely leads to a multinucleate proto-coenocytic proto-organism with flexible, fusible outer membranes and rich internal diversification early on, than to genomically streamlined bacteria-like cells directly. Similar views were stated in general terms before [127,169], but not further elaborated in any detail. Also, by introducing the widening concept of *hierarchical clonality*, I hope to overcome some preconception that "truly" *Darwinian evolution* requires containment and reproducible identity at the level of a rather simple "First Cell" quite early on [65].

Hitherto, the richness of functional RNAs in eukaryotic cells and the assumption of their functional continuity through evolutionary time have been the strongest arguments linking protoeukaryotes to precellular origins directly. Nonetheless, the long-standing myth that prokaryotes must have preceded protoeukaryotes by eons has hardly been disturbed yet in the public mind. Putting focus on the inherently communal nature of primordial evolution in a large organic matrix [106,107] and stressing the importance of spatial substructure in the functional self-organization of such proto-coenocytic super-cells will hopefully strengthen grounds for alternative and, arguably, more realistic interpretations. This is supported by the inference that ecosystems without phagocytosing grazers, which are conspicuously absent among bacteria-like prokaryotic cells, are quite unlikely [205]. It is further underlined by the evolutionary dictum that *K*-type selection, as most prevalent for eukaryotic organisms in general, is of older origin than the highly evolved and specialized means of *r*-selected traits that are most prominent and effective in potentially large, yet rapidly fluctuating populations of prokaryotic cells [200,260,261]. Considering the strength of all these arguments in combination brings the protoeukaryotes-early hypothesis close to ranking as a noteworthy theory-much in contrast to the "*mechanisms founded in unfettered imagination*" [178] that must be invoked by others to save the prokaryotes-early alternative.

As the current essay is primarily concerned with the preliminaries to primal eukaryogenesis, a detailed discussion of how the major lineages of prokaryotic cells can have 'escaped' as such from a communal precellular matrix is beyond the scope of this article. It might well be rewarding to evaluate to what extent the vastly different types of prokaryotic cell wall organization can reasonably be attributed to multiple and partly independent events of micro-cellular escape from a wall-less and still largely communal precellular matrix system and subsequent diverse specialization.

To come back to the epigraph to this outlook section, I do not intend to proclaim certainty about anything I write. Replacing a particular long-standing myth by yet another dogmatic preconception should be none of my concerns. It is entirely up to intelligent readers to judge which set of arguments is going to weigh strongest in the long run. In living up to the spirit of Voltaire [272], however, every upcoming generation of scientists should be prepared to question the merits of traditional preconceptions in the light of whatever circumstantial evidence and/or reasoning might accumulate to warrant alternative solutions. The persistent mantra of "*prokaryotes before protoeukaryotes*" is rife for critical revision. As for the merely rhetorical question, "*The eukaryotic cell arose from prokaryotes just once in four billion years, and otherwise prokaryotes show no tendency to evolve greater complexity.-Why not?*" [275]: this seeming dilemma vanishes into thin air, if bacteria-like prokaryotes never gave rise to the more complex organization of eukaryotic cells in the first place. To be sure, Lane and Martin [275] present a valid cause for unicellular prokaryotes being able to outcompete more complex cell types on about every aspect of primary production. For that matter, only the acquisition of prokaryotic endosymbionts has enabled the modern eukaryotes to survive against this evolutionary pressure. These arguments alone, however, say nothing about the tentative nature of precursory life-like stages where autonomous and efficiently optimized bacteria-like cells did not yet exist.

In conclusion, the incommensurable nature of prokaryotic and eukaryotic cell organization is more deeply rooted in archaic evolution than prokaryotes-early proponents would have us believe. It is in a series of miscible communal states that complexifying organic matter in molecular ecosystems can bridge the formidable gap from a prebiotic Earth to cellular escape of genomically identifiable, autonomous organisms (Figure 1). Well before such liberation of individual propagative cells became possible, the precellular matrix likely attained a quasi-autonomous state of proto-coenocytic organization of highly redundant genomic and structural complexity. From this communal and quasi-syncytial state, protoeukaryotic and prokaryotic cells diverged in accord with complementary, yet antagonistic, evolutionary principles. The *Karyogenic Proto-Coenocyte Hypothesis*, in particular, proposes a novel means of leading up to a *eukaryote-early scenario*, and the general concept of *hierarchical clonality* allows *Darwinian evolution* to commence gradually at various levels. While "*eukaryote evolution has been dominated by large-size (*K*-selection) and redundant features have often been exploited rather than eliminated*" [260], prokaryotic evolution is mainly subject to *r*-selection, favoring small size, high growth rate, specialization, and the elimination of genetic and structural redundancy. Thus, prokaryotic cells rapidly managed to dominate primary production of organic matter, and the residual protoeukaryotic cells only survived by "*taking advantage of prokaryotic activity through predation, symbiosis or habitat selection*" [260].

Complementary, yet antagonistic, principles also prevail at the genetic and structural levels. While eukaryotic cells have retained flexible, fusible outer membranes and stabilized the nuclear division cycle with multiple linear chromosomes, prokaryotes "escaped" both the nuclear confinement of their genes, by making a single circular plasmid big enough to comprise an entire genome, and macro-cellular communality, by perfecting multifunctional outer membranes inside of semi-rigid cell walls. In eukaryotic cells, temporarily fusible outer membranes are still in effect, serving phagocytosis of particulate food items, and/or gametic fusion as a prerequisite for communal sharing of a larger-conspecific-gene pool.

## Note Added in Proof

I apologize to Antoine Danchin for not having incorporated important conceptual findings of his in my discussion. He has expressed views similar to mine on various prebiotic issues, such as the potential roles of uncoded peptides and fusibility prevailing in protocellular communities. Moreover, Danchin recognized that essentially all bacterial genomes carry part of an ancient core set, or "*paleome*", which relates backwards toward the origins of life. This contrasts with large numbers of more recently acquired genes, the "*cenome*", allowing for efficient adaptation to narrow ecological niches [276,277].
